# Community health in a climate disaster: a One Health analysis of workforce exposure in the Eaton wildfire

**DOI:** 10.3389/fpubh.2026.1768565

**Published:** 2026-02-16

**Authors:** Adrienne Martinez-Hollingworth, Natasha Milatovich, Monika Scherer, Zurisadai Inzunza, Linda Kim, Megan Czerwinski, Jeremiah Garza, Elizabeth Kohout, Garrett K. Chan, David P. Eisenman

**Affiliations:** 1 AltaMed Health Services Corporation, Los Angeles, CA, United States; 2 School of Nursing, University of Texas at Austin, Austin, TX, United States; 3 School for Environment and Sustainability, University of Michigan, Ann Arbor, MI, United States; 4 Los Angeles County Department of Public Health, Los Angeles, CA, United States; 5 UCLA Joe C. Wen School of Nursing, Los Angeles, CA, United States; 6 HealthImpact, Oakland, CA, United States; 7 David Geffen School of Medicine at UCLA, Los Angeles, CA, United States

**Keywords:** burnout, community health services, disaster planning, frontline workers, mental health, One Health

## Abstract

**Introduction:**

Climate driven disasters underscore the interdependence of human health and environmental conditions within the One Health framework. This community case study examines the experiences of frontline healthcare workers deployed during the 2025 Eaton Wildfire in Southern California.

**Methods:**

Using a quantitative survey across volunteer staff members serving as frontline healthcare workers (*n* = 113), preliminary data from a phenomenological study of entry-level workers in that group (*n* = 15) and operational insights from the response effort, this paper explores how environmental exposures and community displacement shaped worker wellbeing and workforce capacity. We contextualize these data within the One Health framework.

**Results:**

Most respondents felt motivated to help the community during a crisis (93%) and make a difference in the lives of others (81%), yet more than half reported emotional impact as a significant challenge (53%); deeper qualitative inquiry revealed potential for retriggering due to prior trauma in some workers, including personal experience as a refugee, or history of displacement due to violence. Many identified role clarity concerns (43%), mitigated by scope of practice associated with clinical licensure. While most felt supported (85%), they emphasized the need for peer debriefing and reflection spaces, self-care resources, mental health support, and training for future emergencies.

**Discussion:**

This paper highlights a critical and emerging challenge in climate disaster response: healthcare workers can themselves be disaster survivors, which deeply affects their capacity to respond effectively. Aligning with the One Health principles of holism, collective wellbeing, and reciprocity, the Burnout Dyad is applied to this dual status experience.

**Conclusion:**

Recognizing and supporting these dual status frontline healthcare workers, both responders and survivors, is essential for sustaining resilient health systems in the face of increasing climate crises.

## 1 Introduction

The One Health model recognizes the interdependence of human, animal and environmental health. One Health is a comprehensive and integrated approach that brings together multiple sectors and disciplines to collaboratively promote wellbeing while addressing the essential needs for food, water, energy, and air to support sustainable development (1). One Health has traditionally focused on zoonotic disease, environmental exposures, ecological interconnections. However, the impacts of climate change are increasingly modifying these relationships. Climate-related disasters are events in which hazards such as floods, storms, wildfires, droughts, or extreme temperatures are amplified by the effects of climate change, leading to significant disruptions within communities and resulting in substantial human, economic, or environmental impacts (2). As wildfires, heatwaves, extreme weather events, and poor air quality increases, these disasters highlight the interdependence and interconnectedness among people, animals, and the environment within a community. Each element relies on and contributes to an overall collective wellbeing. For example, pets can support coping among evacuees but pose sanitation challenges in a disaster response context where resources are limited and infectious disease can spread rapidly.

Climate change is reshaping the conditions under which health systems operate and bringing new attention to the strengths and vulnerabilities of people who respond to climate-related emergencies. Frontline healthcare workers play a key role in the resilience of the health systems to provide quality health services in an emergency setting (3). Targeted training, updated policies, and enhanced inter-agency coordination will assist frontline workers to provide health care services to others, while also supporting their direct communities and families (4, 5).

Community health can be described as a collaborative field that brings together multiple disciplines and draws on public health science and evidence-based methods to partner with communities in culturally responsive ways to improve health and life conditions of everyone who lives, works, or otherwise spends time in a particular community (6). Community health workers, medical assistants, nurses, physicians and administrative staff working in safety net healthcare settings frequently reside in neighborhoods that are most affected by environmental hazards (7). Workforce exposure to climate-related disasters refers to both direct or indirect contact with hazards such as wildfire smoke and degraded air quality resulting from job responsibilities or work environments, which may increase the risk of injury, illness, or reduced work capacity (8).

Frontline workers often live in the same high-risk neighborhoods as the patients they serve (9), and experience displacement, smoke exposure, ecological loss and emotional distress at the same time they are called upon to help others (10). As a result, they encounter the impacts of climate-related disasters and displacement both in their professional capacities assisting others and personally, as individuals affected by the same conditions (11).

Dual-status responders are defined as frontline healthcare workers who experience environmental disruption both personally and professionally. This dual status position - responder and survivor - complicates traditional assumptions about the neutrality of frontline healthcare workers. Their personal exposure, emotional burden, and community relationships are inherently connected to the environmental disaster.

**Figure 1:**
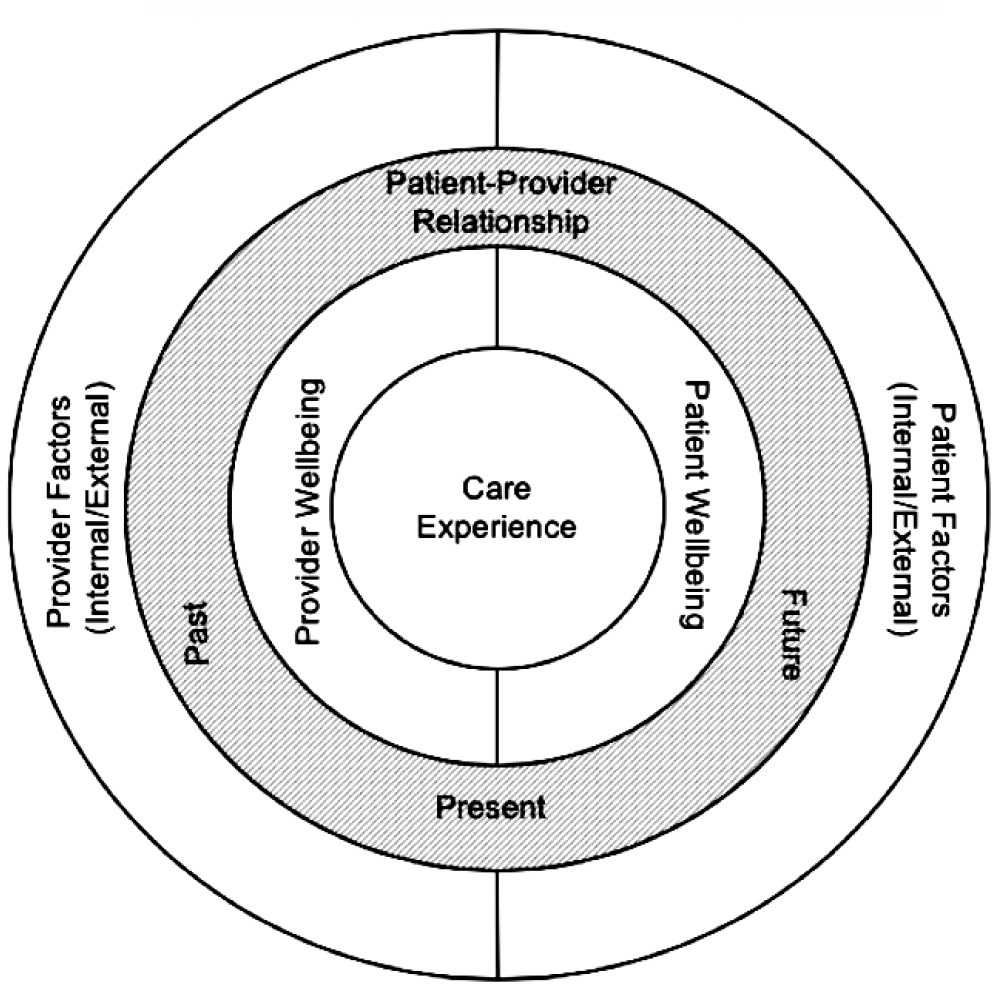
Burnout Dyad Model [(12), p. 287].

The Burnout Dyad [(12), p. 287], a conceptual grounding model (see Figure 1) that describes the complex and reciprocal relationship between patients and providers, offers a lens for understanding how climate events intensify this dynamic. This model views care delivery as a co-constructed encounter shaped by the identities, histories and lived experiences of both patients (evacuees) and providers (frontline healthcare workers at an emergency response). The Burnout Dyad is relevant to CHCs, where workers often share demographic, geographic, and lived experiences with their patient populations. When providers are also disaster affected individuals, the emotional, physical and relational dimensions of their work become more complex. During the 2025 LA Wildfires, the distinction between patient and provider stakeholders overlapped as many first responders of the wildfires had also been displaced and faced the same barriers as those served at Pasadena Convention Center.

When frontline healthcare workers are directly impacted by the disaster, they bring a heightened level of emotional and physical vulnerability into these encounters. A community case study approach effectively illustrates the real-world complexity of a localized climate disaster and how it influences both the personal and professional experiences of frontline healthcare workers. The 2025 Eaton Wildfire in Southern California is a compelling example of this phenomenon. The Eaton wildfire presented a real-world context in which the Burnout Dyad's principles became starkly visible, making workforce wellbeing a central concern within the human health pillar of One Health.

The wildfire began on January 7th, 2025, in Eaton Canyon, a recreational and cultural landmark for surrounding communities in the San Gabriel Valley. As the fire spread across the foothills of Pasadena and Altadena, thousands of residents were forced to evacuate. The 2025 Eaton wildfire burned more than 14,000 acres [(13, 14), p. 913], displaced thousands of residents (15), and compromised air quality across the region (16).

AltaMed Health Services Corporation (AltaMed), one of the largest federally qualified health center (FQHC) systems in the United States, serves predominantly Latino, immigrant and low income communities across Southern California. Many of their ~6,500 employees come from or live in the same neighborhoods as the patients they serve, creating a deep sense of shared identity and community connection. One AltaMed clinic was consumed by the fire and several in the region experienced operational disruptions; hundreds of employees were personally affected by the evacuation alerts, smoke exposure and direct loss. Many frontline healthcare workers were directly exposed to the event, managing uncertainty regarding their own homes and families while simultaneously working long shifts supporting evacuees. This close alignment between personal and professional life shaped the worker experience during this environmental event.

The Pasadena Convention center was designated as the primary evacuation shelter for those impacted by the Eaton wildfire, and AltaMed was one of the first organizations on site to provide healthcare services. The population served in this setting included children, older adults (many of whom were evacuated from one of the many senior care, assisted living, memory care or skilled nursing facilities in the area), individuals with chronic medical needs and families seeking information, reassurance, continuity of care and social services. The frontline workers responding to these needs were AltaMed employees and included medical assistants, licensed vocational and registered nurses, community health workers and educators, care coordinators, behavioral health staff, physicians and administrative volunteers. Many of these frontline workers lived in the same evacuation zones and were experiencing uncertainty about their own safety and resources.

Experiences of the frontline workers illustrate the holistic human health domain within One Health and the interdependence between domains. This community case study examines how one community healthcare organization worked to holistically support the unique strengths and vulnerabilities of dual-status responders to the Eaton Fire, as they supported their community through disaster.

## 2 Methods

The AltaMed frontline worker support initiative developed during the Eaton wildfire response aimed to address the interconnected personal and professional challenges faced by frontline workers. As dual-status responders, frontline workers encountered environmental disruptions both personally and in their professional capacity, both as responders and survivors. Two-hundred and 30 frontline workers who were employed by AltaMed, and volunteered to serve at the evacuation shelter, delivered ~650 clinical and non-clinical services to evacuees. These services ranged from basic first aid and wound care to chronic disease management, medication access and behavioral health services. General assistance including access to regional transportation, replacement clothing and personal care items (toiletries, over-the-counter medications, etc.), and durable medical equipment (canes, walkers, etc. lost in the fire) were also provided by the AltaMed Community Outreach Research Engagement (CORE) team on-site.

The key innovation of this initiative is the support program designed to sustain the health and emotional resilience of these dual-status responders. This program recognized that the workforce was not separate from the disaster but rather embedded within it. Supporting dual status responders required an approach attentive to their overlapping roles, vulnerabilities and recovery needs. Although AltaMed supported volunteer programs across several recovery events between January and November 2025, the present case study centers exclusively on the immediate and 3-month period following the Eaton response in order to provide a clear and focused analysis of program design and workforce experience.

This community case study reflects a real-world program and emphasizes a critical analysis of an initiative aimed at enhancing the health and wellbeing of frontline workers during a climate-related disaster (17). The theoretical framework is based on principles of community engagement and community-based participatory research (CBPR) which positions communities as equal partners in identifying issues and equitably involves community members to collaboratively develop interventions to improve health outcomes (18, 19). The case study utilized a descriptive design which allowed for a detailed examination within the real-life context. The survey was distributed to 230 frontline workers serving at the evacuation center, with 113 participants completing the survey. Data was collected anonymously with no identifiers saved, ensuring that individual responses could not be linked to participants. No identifying information or details linking responses to specific departments were collected, ensuring participant confidentiality and protecting them from potential retaliation or employment-related risks.

The survey instrument (Qualtrics survey) was distributed via email prior to the debrief sessions with five questions, multiple choice with selecting all that apply options. Due to the timeliness of the sessions, the survey questions initial aim was for disaster preparedness quality improvement and employee mental health wellbeing and did not derive from existing validated surveys. The survey domains include motivations for volunteering at the wildfire evacuation center, rewarding and challenging aspects of the role, how supported they felt in the role, what support or resources would be most helpful to them, and what skills or knowledge would be helpful to have prior to responding to the wildfire disaster. For data analysis, quantitative responses were analyzed using descriptive statistics, while qualitative data were assessed through open coding procedures.

Additional data was collected via surveys following debrief sessions, qualitative notes across organization meetings and via a phenomenological study of entry-level workers (*n* = 15), and a grounded theory methodology (GTM) study of providers and leaders who served at the site (*n* = 16), which allowed for deeper exploration of findings in the initial survey, as well as the development of a paradigm and contrast case. Drawing from Charmaz's (20) constructivist grounded theory approach, data analysis emphasized the co-construction of meaning between participants and the study team, while remaining attentive to power dynamics, organizational structures, and the socio-ecological context. GTM offers a systematic approach to analyzing qualitative data with the goal of generating a conceptual model grounded in participants' lived experiences, social contexts, and interactions. Furthermore, in grounded theory and participatory implementation research, the trustworthiness of findings is established through systematic, iterative, and transparent processes that uphold rigor across all stages of theory construction and application. Maslow's (21) Hierarchy of Needs framework is used to explore how unmet foundational needs may constrain role performance and wellbeing during disaster deployment. The Socio-Ecological Model broadens the perspective of the case study lens by expanding the focus from individual and interpersonal factors to multiple interconnected levels of influence, including organizational, community, and policy factors to understand how these various levels collectively shape how people respond to and recover from adverse events, including climate-related disasters (22, 23).

Interpretive phenomenology is based on Heidegger's (24) philosophical work that informed Benner's Interpretive Phenomenology (25) which drove our secondary phenomenological study of entry-level workers via a focus on their lived experiences and how they consciously perceive and make sense of the world around them. This approach is descriptive, rather than explanatory and in this work is used to explore findings from the large-scale survey across all volunteers. The grounded theory semi-structured interview guide was designed to follow-up on data collected from the entry-level workers from the perspective of administrator, nurse and physician leaders. Full GTM analysis is presented elsewhere.

The quantitative survey was classified as a quality improvement project and IRB review was not required for these anonymous surveys; however, IRB was obtained for phenomenological exploration (WCG IRB#1393995). Some of these quantitative data were previously presented elsewhere, including community-facing presentations, but are being highlighted here in the context of dual identity within the One Health framework and expanded via the qualitative, phenomenological findings.

**Figure 2:**
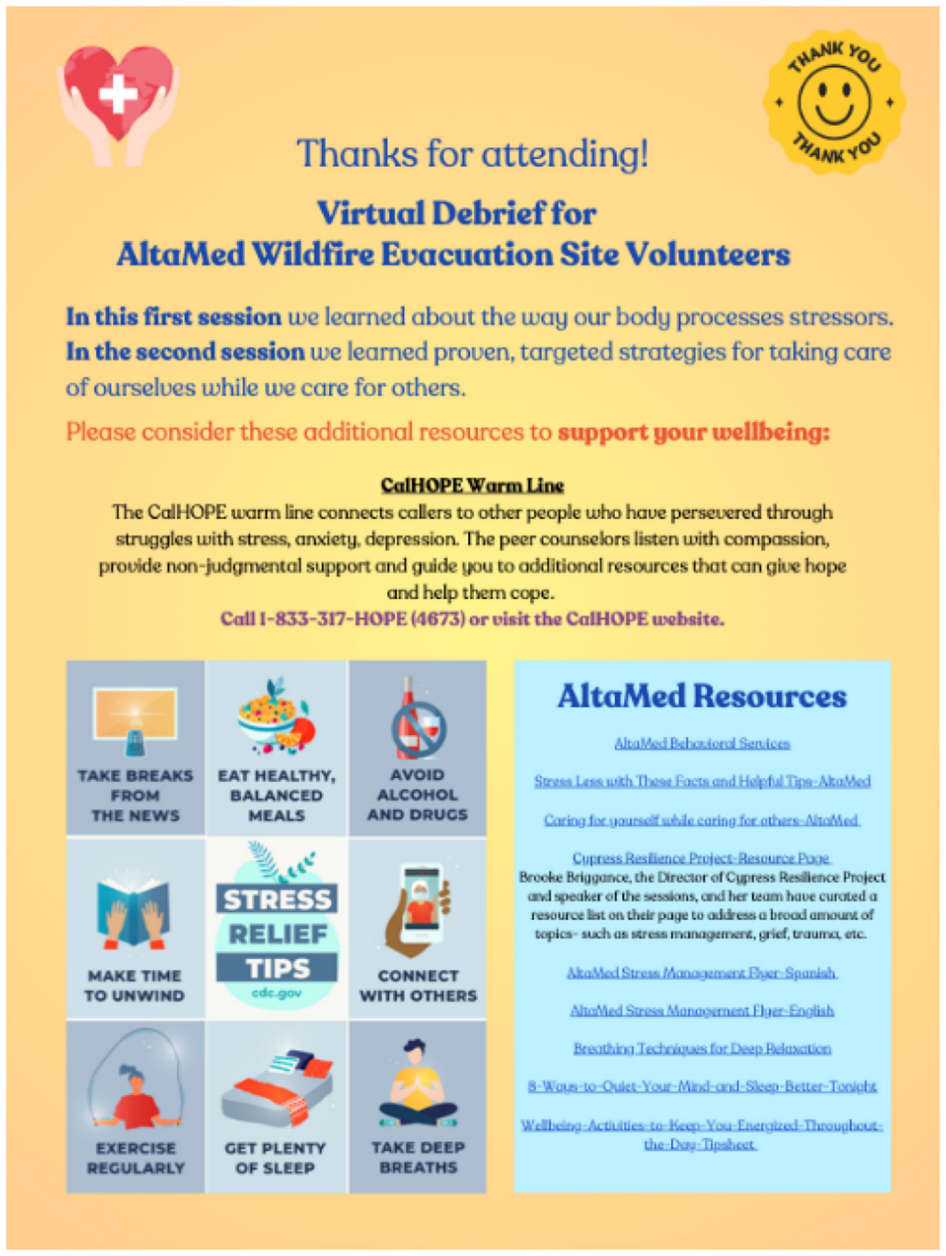
Flier for post-disaster relief resources disseminated to frontline workers.

To further support ongoing recovery, the research, implementation science and evaluation team created a comprehensive resource page that consolidated stress management tools, behavioral health services, guidance on wildfire smoke exposure and information relevant to disaster recovery. This resource flier was disseminated to post-disaster frontline workers (Figure 2). It was intended to support workers throughout the weeks and months following the event, recognizing the emotional recovery often extends far beyond the initial response period.

## 3 Results

Across all data, we repeatedly see the interaction of altruism, identity, environmental chaos, and structural inequities producing unique emotional, professional, and ethical stressors. These outcomes are pronounced in dual status responders. These are the volunteers who both serve in the disaster response workforce and live or have a personal connection to the impacted areas.

Staff approached this work with an extraordinary level of commitment. Per the post-service survey (*n* = 113), nearly all who participated were motivated by a desire to help the community during the crisis, and many were driven by the hope of making a meaningful difference. Others reflected on the value of working alongside colleagues and the opportunity to gain new skills during an unprecedented event (Table 1).

**Table 1:** Post-service survey results (*n* = 113).

**Category**	**Details**	**Percentage (*n*)**
**Motivations for…**		
Desire to help community	“I want to help during a crisis”	93% (*n* = 105)
Making a difference	“I want to make a difference in people's lives”	81% (*n* = 91)
Collaboration	“I want to collaborate with an amazing team”	56% (*n* = 63)
Learning	“I want to learn new skills and gain experience”	46% (*n* = 52)
**Challenging aspects…**		
Emotional impact	“I experienced an emotional impact from the work”	53% (*n* = 60)
Role clarity	“I felt a lack of role clarity”	43% (*n* = 49)
**Sense of support…**		
Very supported	“I felt very supported”	49% (*n* = 55)
Supported	“I felt supported”	36% (*n* = 41)
**Identified support for…**		
Reflection	“I felt there were opportunities to debrief or reflect with peers”	43% (*n* = 49)
Resources	“I felt there were self-care resources for recovery”	32% (*n* = 36)
Mental health	“I felt there was access to mental health support (e.g., counseling, resilience sessions)	23% (*n* = 26)
Training and resources	“I felt there was training or resources for future disaster response efforts”	56% (*n* = 63)

In interviews, respondents reported a deep sense of service and moral obligation. One individual stated: “It was blessing to be part of a team that wanted to help.” Another said, “if that's the assignment, just sign me up like I'll be there.” (CHW 7). Another said: “I feel like human connection can really bring you that sense of like positivity, good energy, to believe in yourself and to also believe in the community and what we're going through”. (CHW 12)

They also reflected on identity-based connection to affected neighborhoods and colleagues who were impacted:

*Telling my family that I feel so bad for all these women who are going through it …* (speaking about colleagues who live and work in the area) *and they don't have a family member here, so I feel like that those stories really impacted me*. (CHW 12)

In this instance, an employee was called upon to be that “default” family and support system for a colleague.

Many were hopeful for the opportunity this event created for their professional growth and learning, despite feeling unprepared in advance for such events:

*There were times that I might have not felt comfortable doing something like that, but because it was part of my job and because I was there to do that job… For me, it was surprising that, oh my god, I did that, and it was ok, and I felt safe… like I wouldn't do that on a normal day, but at the same time I was very proud of myself for doing it*. (CHW 13)

Another respondent reflected:

*To gather resources to be helpful, aside from everyone's emotional reaction around me, I felt like I was the go-to person because you know being in healthcare and just being proactive, I just felt overwhelmed because I didn't know how to really react to that*.

Staff reported experiencing significant emotional stress related to the demanding nature of their work and their personal experiences with the fire. This aligns with Maslow's (21) Hierarchy of Needs Framework, suggesting that unmet needs may have impaired their ability to perform effectively. More than half identified the emotional impact as the most challenging part of their service (53%): “…the mental toll that it had of those stories and those people… it was just very sad.” (Licensed Vocational Nurse [LVN] 2) Another relayed that he had evacuated his family before coming to serve on-site at the evacuation center. He mentioned that the work was distracting him from the fear and uncertainty that his family might have lost all their possessions. One of the employees remembered:

*Some of our people* (employees) *were there like they were being evacuated from their houses. I'm like, what are you doing here? You shouldn't be here, you know like you're taking care of the same people that you're probably gonna be in in a couple of days or something, you know, I don't know*. (Registered Nurse [RN] 12)

This dual strain underscored the importance of supporting workers in a manner consistent with the principles of the Burnout Dyad, which emphasizes that care providers do not leave their identities or personal histories (or present reality including housing situation) at the clinic door. During a climate disaster, their personal exposure deepens the interdependence between patient and provider experiences. In line with the SEM framework, multiple levels of influence shaped how individuals responded and recovered, clarifying how they reintegrated into the workplace while managing residual impacts of the climate-related disaster:

*How do they go back to work you know the next day or a week later thinking about all that, you know, I still think of the patient that I had who lost her home and her insurance company had cut off her fire (coverage) you know and so…* (Registered Nurse [RN] 12)

Another said:

…*like it's - it's in your head, like you overthink it and like it was kind of like harder to sleep at night just thinking about all those people but nothing that I can (do)… like nothing that was affecting my life and my work the next day… (but still felt impacted)*. (CHW 12)

The emotional toll was evident in employees' reactions during the immediate period following closure of the center. This individual continued:

“I came back home, and I like I remember one morning I woke up, I was crying like I wasn't needed in the morning at (evacuation center), but I was needed after 12 (in normal clinic role), but I was just crying so much. I remember.” They added: “The mental toll that it had on those stories and those people like I just, it was just very sad, yeah.” (CHW 12).

Participants described the value of sharing their experiences with peers who both understood the demands of the work and the realities of the wildfire devastation. In response to these challenges, AltaMed implemented a virtual debrief series facilitated by the Cypress Resilience Project with session one held on January 24, 2025 and session two held on January 31, 2025. These sessions provided education on stress physiology, strategies for managing emotional challenges, and opportunities for guided reflection. They emphasized how shared identity and collective processing can facilitate more effective recovery following environmental trauma.

## 4 Discussion

The anonymous survey provided feedback on frontline workers' experiences serving at the evacuation center. Respondents identified the need for additional opportunities to reflect with peers, access to self-care resources, mental health support, and training for future response efforts. These findings were consistent with the experiences described during debrief sessions and reinforced the ongoing need for structures that acknowledge the complex reality of dual status responding.

The Eaton Wildfire illustrates how the climate crisis has altered the nature of disaster response in community health settings. Frontline healthcare workers may be unable to remain as neutral participants in response efforts as they are often members of the affected communities sharing identical landscapes, neighborhoods, and social conditions that climate disasters disrupt. Their proximity to environmental harm creates a dual identity, as individuals personally affected by the disaster and as healthcare professionals committed to supporting others (26).

This dual status response reveals potential implications for the One Health model. The framework's human pillar must include not only patient populations but also the wellbeing of the workforce that sustains the health system through crisis. Workers' emotional and physical wellbeing is a factor in their capacity to deliver effective care, maintain system functioning, contribute to community resilience, and ultimately return to their pre-disaster roles. When the workforce is strained, the health system becomes vulnerable.

The Burnout Dyad offers a way to understand this phenomenon. This model asserts that patient and provider experiences are intertwined and that care encounters are shaped by the identities and inner states of both participants. The Eaton response demonstrated how climate disasters amplify these dynamics. Workers' emotional distress, concerns for their own families, and grief over ecological loss intersected with their responsibility to support evacuees. These overlapping pressures influenced how they experienced the response, how they engaged with patients (evacuees) and what they needed to recover and return to work.

The frontline worker support initiative described in this community case study offers framing and practical strategies that could strengthen workforce resilience during future climate events. The virtual debrief sessions created structured spaces for reflection, emotional processing and peer connection. The resource hub provided readily accessible guidance and recovery tools. Organizational acknowledgment of dual status responding helped validate the experiences of workers who were balancing their professional obligations with their own personal needs.

Several lessons emerge for future applications. Workforce preparedness training should incorporate discussions of dual status responding and clarify expectations for staff during climate events. Role clarity must be prioritized to reduce anxiety and support a sense of control during unpredictable circumstances. Emotional recovery should be built into the response timeline rather than offered as an optional afterthought. Community health systems must acknowledge that climate driven disasters will increasingly affect their workforce directly, making workforce wellbeing a critical component of any comprehensive One Health strategy (Table 2).

**Table 2:** Table of recommendations for implementing a comprehensive One Health strategy.

**Recommendation**	**Description**
Virtual debrief sessions	Structured spaces for reflection, emotional processing and peer connection
Resource hub	Readily accessible guidance and recovery tools
Organizational acknowledgement of dual status responders	Assists to validate the experiences of workers who were balancing their professional obligations with their own personal needs
Workforce preparedness training	Incorporate discussions of dual status responding and clarify expectations for staff during climate events
Role clarity	Clear roles to help reduce anxiety and provide support and sense of control during unpredictable circumstances
Emotional recovery time	Allocated time built into the response timeline rather than offered as an optional afterthought

Policy recommendations include addressing role clarity and recognition of dual-status responders in local, state, and organizational emergency plans. It is also important to incorporate structured emotional support into response operations. Additionally, emergency preparedness protocols should encompass provisions for workforce wellbeing and the risks associated with dual-status roles. Including scenario-based exercises specific to climate-related disasters will enable frontline workers to practice new procedures in a controlled environment, thereby reducing the need for on-the-spot learning during a disaster response. Furthermore, health systems should integrate workforce wellbeing indicators into their climate adaptation strategies to effectively monitor disaster preparedness metrics.

### 4.1 Limitations

This community case study reflects the implementation of a practice oriented program, rather than a formal research study. Insights are based on the perspectives of volunteers who chose to participate in debrief sessions or complete surveys, and therefore may not reflect the experiences of all responders. Quantitative data were collected for internal quality improvement rather than generalizable research; a validated instrument was not used. The quantitative survey instrument was developed with the primary aim of quality improvement purposes, therefore the instrument used was not subject to validation. As a result, there is a limitation for the reliability and comparability of the quantitative findings from this paper. Additionally, the case study focuses exclusively on Eaton Wildfire response, despite the concurrent wildfire event occurring in Los Angeles County, the Palisades Wildfire. Generalizability to other climate disasters or settings may be limited due to unique geographic, demographic, and event-specific factors. The use of a convenience sample of the volunteer frontline workers (*n* = 113) could introduce selection bias, as those who volunteered to serve as a frontline worker at the evacuation center may have different motivations. Data was collected through self-reported surveys and operational notes, which may be influenced by recall bias and the potential social desirability bias. While confidentiality was maintained, the voluntary nature of the survey may have discouraged some individuals from sharing concerns. The program was implemented within a single community health system. Despite its substantial reach in the region, the organizational culture, resources and population characteristics may differ in other environments and similarly limit generalizability. If there is greater demographic concordance between responders and evacuees within an organization, the dual role of participants as both responders and evacuees introduces complex dynamics that may not be fully captured through this case study and the survey instruments alone. This highlights the need for future studies using longitudinal and mixed-methods approaches across broader populations and other climate-related disaster contexts.

## 5 Conclusion

Climate change and the increasing prevalence of climate-related disasters is challenging the persistent assumption of frontline and healthcare responder neutrality. Individuals often influence, and are influenced, by their lived environment. Holistic, trauma-informed support for communities and frontline responders has often been lacking, especially in disaster contexts. Increasing dual-status responder experiences amidst accelerating climate change, provides urgency and opportunity to do better. Utilizing the Burnout Dyad Model and its congruence with the principles of interdependence, holism, and reciprocity embodied in the One Health Framework, this case study shows how one community healthcare organization worked to do better amidst wildfire disaster.

## Data Availability

The raw data supporting the conclusions of this article will be made available by the authors, without undue reservation.
